# Supplementary material for: Characterizing cell-type spatial relationships across length scales in spatially resolved omics data
Source: Nat Commun. 2025 Jan 3;16:350. doi: 10.1038/s41467-024-55700-1 (PMC11699133; doi:10.1038/s41467-024-55700-1)
Supplement: Supplementary file 3 — Supplementary Software 1 [file 41467_2024_55700_MOESM3_ESM.zip › CRAWDAD/plots/supplementary/S7_cluster_exp_XXCD_annot.pdf]

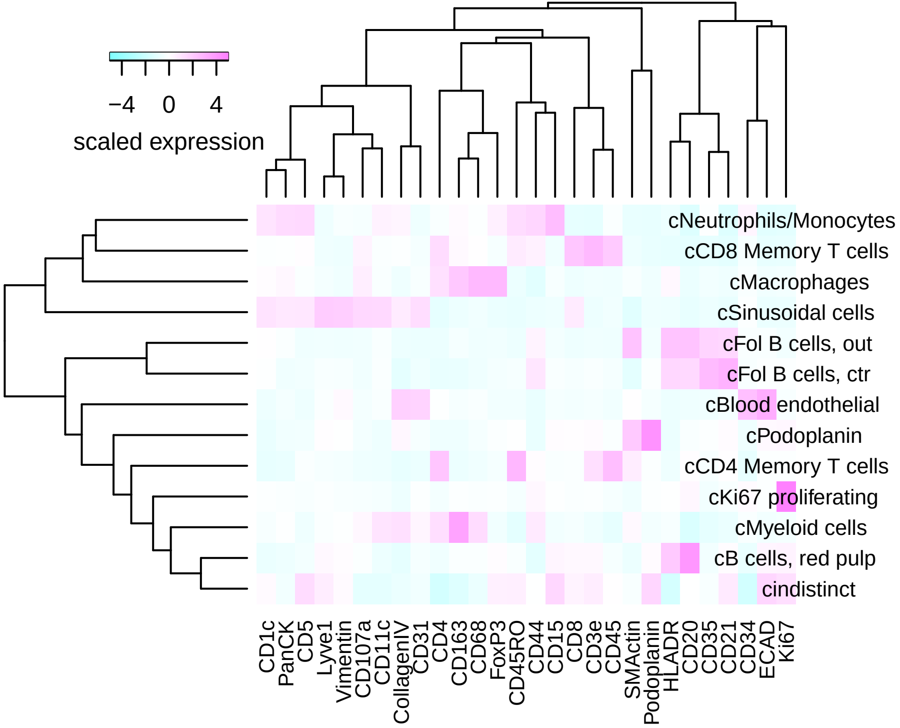
−4 0 4
scaled expression
cNeutrophils/Monocytes
cCD8 Memory T cells
cMacrophages
cSinusoidal cells
cFol B cells, out
cFol B cells, ctr
cBlood endothelial
cPodoplanin
cCD4 Memory T cells
cKi67 proliferating
cMyeloid cells
cB cells, red pulp
cindistinct
CD1c
PanCK
CD5
Lyve1
Vimentin
CD107a
CD11c
CollagenIV
CD31
CD4
CD163
CD68
FoxP3
CD45RO
CD44
CD15
CD8
CD3e
CD45
SMActin
Podoplanin
HLADR
CD20
CD35
CD21
CD34
ECAD
Ki67
